# Laparoscopic Gastric Band Placement in Combination With Sleeve Gastrectomy for Advanced Weight Loss: A Case Report

**DOI:** 10.7759/cureus.25246

**Published:** 2022-05-23

**Authors:** Sataj Johnson, Drew Mazurkiewicz, Veronica Velez, Avery Richardson, Frederick Tiesenga

**Affiliations:** 1 General Surgery, Saint James School of Medicine, Park Ridge, USA; 2 General Surgery, Windsor University School of Medicine, Frankfort, USA; 3 General Surgery, Borough of Manhattan Community College, New York City, USA; 4 General Surgery, West Suburban Medical Center, Chicago, USA

**Keywords:** laparoscopic adjustable band, bariatric surgery, sleeve gastrectomy, weight loss, obesity

## Abstract

Laparoscopic adjustable gastric banding (LAGB) over laparoscopic sleeve gastrectomy (LSG) is a rare bariatric surgery. The objective of this case report is to add to the limited literature regarding this procedure. LAGB plus LSG is an effective bariatric procedure for patients that desire additional weight loss who do not qualify for or want gastric bypass. A 39-year-old African American Female with a pertinent past medical history of morbid obesity, gastroesophageal reflux disease (GERD), sleep apnea, and chronic back pain presents for gastric bypass consultation in 2020. The patient had a prior bariatric surgical history of gastric band placement (2014), removal (2016), and LSG (2018). The patient was unsatisfied with her weight loss and pursued a consultation for gastric bypass. Upon further investigation, gastric bypass was not indicated for the patient due to low BMI. The patient was presented with the option of LAGB over LSG for increased weight loss and improvement or resolution of comorbid conditions. The patient appreciated the physicians' recommendation, and on June 3, 2021, a LAGB was placed over the prior sleeve gastrectomy. No complications occurred preoperatively or postoperatively. The patient continues to adhere to diet and exercise instructions, and at her most recent follow-up, she reported resolution of GERD symptoms and had a reduced BMI of 33.2. It is important to note that the long-term outcomes of this procedure are yet to be fully discovered due to the rarity of the surgery and some literature deficiencies. Overall, LAGB, in combination with LSG, is a safe and effective procedure for increasing weight loss, improving quality of life, and decreasing mortality for patients for whom the surgery is indicated.

## Introduction

As the rates of obesity rise in the United States (prevalence of 30.5% from 2000 to 2017 to 41.9% from 2017 to 2020), so do the rates of death due to this preventative cause [1]. When it comes to preventable death in the United States, obesity ranks at the top of the list in second following only smoking. Recent advancements in bariatric surgery are leading the fight against the obesity pandemic. Although bariatric surgery is not new, with the first jejunoileal bypass procedure being performed in the 1960s, recent advancements in procedure techniques have made the surgeries safer and more effective [2]. Currently, there are three widely accepted bariatric procedures. The first of these three popular procedures followed soon after discovering the jejunoileal bypass and was termed gastric bypass. The most common gastric bypass procedure performed is the Roux-en-Y gastric bypass. Before introducing less invasive methods, Roux-en-Y gastric bypass was the procedure of choice for 60-70% of all bariatric surgeries [3]. The first of the two newer procedures is the laparoscopic adjustable gastric band (LAGB). The first LAGB procedure was successfully performed in 1993 and was approved by the FDA as a weight loss procedure in the United States in 2001. The second procedure that replaced the gastric bypass method is currently the most common bariatric procedure termed laparoscopic sleeve gastrectomy (LSG). The first LSG was performed in 1999 and quickly became the most popular weight loss surgery by the late 2000s, with indications for LSG being published in 2008 [2]. The type of bariatric surgery used is based on the patient's preference, but each technique does have its own indications. In rare cases, as in the case we present today, the patient elected to have a gastric sleeve procedure performed and, after minimal weight loss goals were reached, elected to have a gastric band placed on top of the gastric sleeve. Current literature mentions LAGB as a revisional procedure for Roux-en-Y gastric bypass. However, this case report will aid in the literature deficiencies of cases of LAGB over the gastric sleeve. An informed consent form was obtained from the patient for this case study to be completed.

## Case presentation

**Figure 1:**
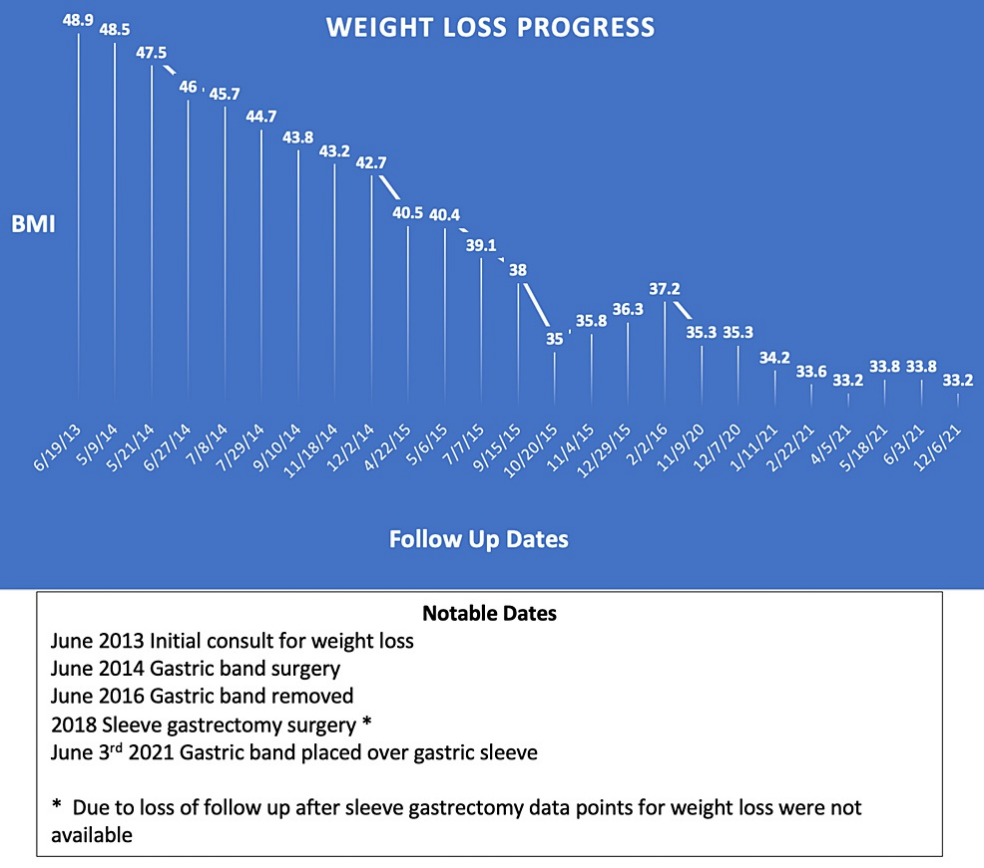
Preoperative and postoperative BMI values.

A 39-year-old African American female with a pertinent past medical history of morbid obesity, prediabetes, back pain, joint pain, sleep apnea, and gastroesophageal reflux disease (GERD) presents for gastric bypass consultation. The patient's family, social and occupational history are non-contributory. The patient took metformin in the past for prediabetes and phentermine for weight loss but no longer requires the metformin. The patient has no known allergies. Past surgical history consists of salpingostomy for ectopic pregnancy, laparoscopic gastric band (2014), laparoscopic cholecystectomy (2015), gastric band removal (2016), and sleeve gastrectomy (2018). The patient underwent multiple band adjustments due to inadequate restriction, vomiting, stomach discomfort, new-onset GERD, and continuous GERD symptoms. The patient's BMI went from 48.9 to 37.2 during her time with the gastric band. Due to unsatisfactory weight loss, the patient decided to undergo sleeve gastrectomy. The patient recommitted to positive lifestyle changes and dietary habits but was still not satisfied with her weight loss. Her BMI went from 37.2 to 35.3 with the gastric sleeve. In November 2020, the patient presented to the clinic desiring a gastric bypass. The patient was informed that she might not be a candidate for gastric bypass due to her low BMI (35.3). The patient understood the risks, benefits, and indications for gastric bypass, and additional options were presented to her. The safest and most effective alternate option presented to the patient was a gastric band over sleeve. She appreciated the recommendation and decided to move forward with the gastric band over sleeve procedure to accomplish her goal weight. Diagnostic laparoscopy, adhesiolysis, and the gastric band over sleeve procedure were performed in June 2021 with no complications intraoperatively or postoperatively. The patient agreed to continue to follow up, adhere to diet restrictions, and continue taking phentermine and calcium + vitamin D tablets. At the patient's most recent follow-up, her GERD symptoms resolved, and her BMI was down to 33.2 (Figure 1).

## Discussion

The laparoscopic band was first introduced in a non-adjustable form in the 1970s, with the more successful adjustable band following in 1985 [2]. The LAGB reached peak popularity in 2008 but declined soon after due to the LSG. Indications for LAGB include a BMI of 40, a BMI greater than 35 with obesity-related comorbidities such as obstructive sleep apnea or diabetes mellitus type 2 [2]. Patients can also be considered for LAGB if their BMI is 30-35 with an obesity-related comorbid condition. Patients are only considered for LAGB once they have failed nonsurgical weight-loss strategies, such as diet and exercise. Contraindications for LAGB include but are not limited to pregnancy, uncontrollable coagulopathy, and inability to tolerate general anesthesia [2]. LAGB has the lowest mortality of all other bariatric procedures, with a rate of 0.02%-0.01% [2]. Although LAGB has the lowest mortality rate, it does not come without complications. Complications of LAGB include gastric prolapse, otherwise known as a slipped band, device malfunction, band erosion, and band obstruction. A slipped band is characterized as the lower stomach herniating through the band superiorly, causing the band to move from its original diagonal position to a horizontal position. Patients with a slipped band can present sudden onset abdominal discomfort and reflux symptoms. Slipped bands can be adjusted via deflation, and further procedures can be considered based on patients' lack of clinical improvement. Band erosion has a low incidence of 1-2% due to abdominal pain and port site infections [2]. Device malfunctions consist of port dislodgement, which will deem the port inaccessible, tube kinking, and device leak. Band obstruction due to an over-inflated band is the most common cause [2]. Band obstruction leads to esophageal and gastric pouch dilation, which can also be treated with band deflation or removal, if necessary [2]. LAGB was once the preferred bariatric surgery method accounting for 42.3% of all bariatric surgeries worldwide in 2008 [2]. LAGB shows adequate weight loss and is still widely indicated.

LSG is a procedure that consists of resecting the greater curvature of the stomach leaving it more narrow. LSG was first used as a step in a staged gastric bypass surgical plan for morbidly obese patients [4]. Whether LSG is used as a definitive or staged weight loss strategy, its impact on obesity is via restriction and hormone modulation [4]. This procedure is a preferred bariatric surgery method for patients who meet the criteria and desire a more permanent option compared to LAGB. Bariatric surgery has the main benefit of weight loss but an emphasis is also placed on the reduction of comorbid conditions. In addition to a 33%-90% excessive weight loss percentage over six to thirty-six months, LSG also proved to resolve or reduce comorbidities such as diabetes, hypertension, GERD, degenerative joint disease, and sleep apnea [4]. LSG is a low-risk procedure with a low likelihood of complications. Potential complications are staple line leakage which carries a risk of 1.17%, and internal bleeding (3.57%). The procedure mortality rate is only 0.3% [4]. Some other advantages of LSG are the lack of intestinal anastomosis, no implantation of a foreign body, and reduced malabsorptive side effects [4]. Since most bariatric procedures have the same relative indications and contraindications, deciding between them relies heavily on patient weight loss goals and preferences. Patients who have weight loss goals that are not met with LSG are then considered for other bariatric procedures.

The most invasive weight loss procedure, gastric bypass, consists of creating a small pouch from the stomach and connecting the newly created pouch directly to the small intestine. Swallowed food bypasses most of the stomach and the first part of the small intestine. While malabsorptive procedures such as gastric bypass are more effective in contributing to weight loss than primarily restrictive procedures such as LAGB, patients are at increased risk for nutritional deficiencies [5]. Due to the increased risk of nutritional deficiencies, patients who undergo gastric bypass must abide by supplemental regimens. This surgical procedure is commonly done only after trying to lose weight with nonsurgical interventions or less invasive procedures. Patients with a BMI of 40 or greater are premier candidates for this surgery [5]. In addition, patients must possess the willingness to make permanent lifestyle changes. Participation in long-term follow-up appointments to monitor nutrition, behavior, and comorbid conditions is crucial to positive future outcomes. Like any invasive procedure, gastric bypass has risks and complications, including malnutrition, stomal stenosis, dumping syndrome, and peritonitis [5].

As previously discussed, patients unsatisfied with their weight loss after enduring any bariatric procedure can be considered for another, as the case was with our patient. One of those options is a LAGB over LSG. This surgery has proven efficacious in continued weight loss and resolution or improvement of comorbid conditions. One study showed that the percentage of excess weight loss with LSG alone was 32.2%, and the percentage of excess weight loss of LSG plus LAGB was 57% [6]. Patients who underwent this procedure had a reduction in BMI from 43.9 with LSG alone to 37.6 with LSG plus LAGB [6]. Similarly, our patient had a reduction in BMI from 35.3 with LSG alone to 33.2 with LAGB plus LSG. This procedure is indicated for patients who no longer meet the BMI criteria for gastric bypass or prefer a less invasive procedure but desire additional weight loss. Patients experience the advantage of increased weight loss without undergoing a highly invasive operation, with increased long-term effects such as malabsorption and nutritional deficiencies. Therefore, special consideration should be given when providing patients with bariatric surgery options and information. The decision ultimately comes down to satisfying patients' weight loss goals and resolving comorbid conditions, conclusively improving patients' quality of life.

## Conclusions

A laparoscopic adjustable band is understood to be the least invasive bariatric procedure. LAGB can be used in addition to LSG if prior treatment fails. The objective is to satisfy patients' unsatisfactory weight loss by using a combination of restriction and minimal absorption methods. It is always essential to consider surgical indications and risk versus benefit ratios. Excessive weight loss and resolution of comorbidities are variable with bariatric surgeries. Continued and preserved weight loss depends on patient education and execution regarding diet, exercise, and vital bariatric follow-up evaluation. 
